# From Soft Lithography to 3D Printing: Current Status and Future of Microfluidic Device Fabrication

**DOI:** 10.3390/polym17040455

**Published:** 2025-02-09

**Authors:** Jingjing Xu, Michael Harasek, Margit Gföhler

**Affiliations:** 1 Institute of Engineering Design and Product Development, Technische Universität Wien, 1060 Vienna, Austria; margit.gfoehler@tuwien.ac.at; 2 Institute of Chemical, Environmental and Bioscience Engineering, Technische Universität Wien, 1060 Vienna, Austria; michael.harasek@tuwien.ac.at

**Keywords:** 3D printing, additive manufacturing, soft lithography, microfluidic engineering

## Abstract

The advent of 3D printing has revolutionized the fabrication of microfluidic devices, offering a compelling alternative to traditional soft lithography techniques. This review explores the potential of 3D printing, particularly photopolymerization techniques, fused deposition modeling, and material jetting, in advancing microfluidics. We analyze the advantages of 3D printing in terms of cost efficiency, geometric complexity, and material versatility while addressing key challenges such as material transparency and biocompatibility, which have represented the limiting factors for its widespread adoption. Recent developments in printing technologies and materials are highlighted, underscoring the progress in overcoming these barriers. Finally, we discuss future trends and opportunities, including advancements in printing resolution and speed, the development of new printable materials, process standardization, and the emergence of bioprinting for organ-on-a-chip applications. Sustainability and regulatory frameworks are also considered critical aspects shaping the future of 3D-printed microfluidics. By bridging the gap between traditional and emerging fabrication techniques, this review aims to illuminate the transformative potential of 3D printing in microfluidic device manufacturing.

## 1. Introduction

Microfluidic devices, often referred to as lab-on-a-chip systems, have revolutionized numerous fields of science and engineering by enabling the precise manipulation of small fluid volumes through channels with dimensions typically ranging from tens to hundreds of micrometers [1]. The development of microfluidics has provided unprecedented opportunities in various applications, including biomedical diagnostics, chemical synthesis, and environmental monitoring [2]. One of the main advantages of microfluidic technology is its ability to integrate multiple laboratory functions onto a single chip, thereby reducing the required sample volumes, shortening analysis times, and lowering reagent consumption [3,4]. This miniaturization not only enhances the efficiency of analytical processes but also opens up new possibilities for point-of-care testing and personalized medicine [3,5,6].

Additionally, the ability to precisely control the microenvironment within these devices facilitates the study of cellular behaviors and the development of organ-on-a-chip systems, which are invaluable for drug screening and disease modeling [7]. Considering physics and materials science, microfluidic platforms enable the study of fluid dynamics at small scales, offering insights into phenomena such as laminar flow, diffusion, and surface tension effects.

**Figure 1:**
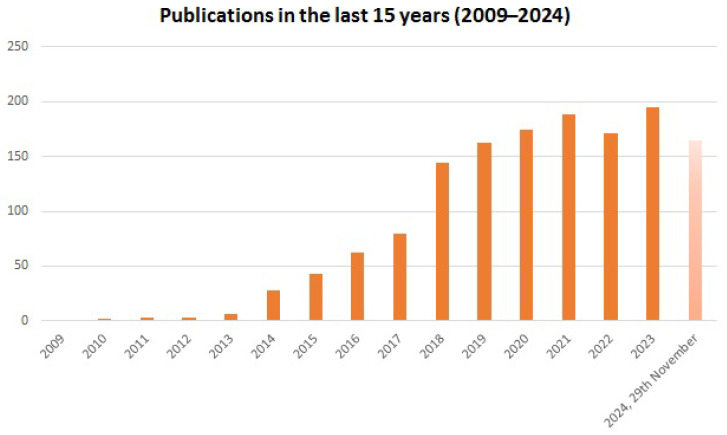
Publications with the topics “microfluidic” and “3D printing” from 2009 to 2024. Numbers were collected from the Web of Science platform by searching the aforementioned topics and refining the publication years from 2009 to 2024.

Advances in fabrication techniques, including soft lithography and 3D printing, have expanded the accessibility and versatility of microfluidic devices, allowing for the creation of complex, multi-functional systems tailored to specific research needs. While 3D printing is widely used in other industries, its application in the production of microfluidic chips is still limited, with soft lithography remaining the most commonly employed technology due to its reliability, precision, and ease of use [8]. To date, the vast majority of chips are molded by soft lithography using polydimethylsiloxane (PDMS), a material which saw great success thanks to its multiple key properties, such as its biocompatibility, elastomeric behavior, transparency, gas and water permeability, and its relatively low cost [4,8,9]. On the other hand, 3D printing is struggling to establish itself in the sector as, to date, there is still no material with characteristics comparable to those of PDMS, and the produced devices lack high printing resolutions [8]. Despite these limitations, recent years have seen rapid advancements in 3D printing, consistently introducing innovative solutions which push it closer to becoming the leading technology in microfluidic production. Figure 1 illustrates this progress, showing a significant increase in publications on 3D-printed microfluidic devices over the past 15 years.

As the field of microfluidics continues to evolve, promising to bring significant innovations in various scientific disciplines, it is also important to continuously update its production techniques, which must be able to reproduce increasingly complex models. This review aims to provide a comprehensive overview of the most-used 3D printing technologies, and by examining them, we seek to highlight their respective strengths, limitations, and potential future directions in the context of microfluidic device development.

## 2. Soft Lithography

Soft lithography is a versatile and cost-effective technique for fabricating micro- and nanoscale patterns, being widely utilized in microfluidics [10], materials science [11,12], and biotechnology [13]. Introduced in the early 1990s by George M. Whitesides et al., this method emerged as an innovative alternative to traditional photolithography, offering a simpler, more accessible approach to pattern generation [14].

At its core, soft lithography employs an elastomeric material, typically polydimethylsiloxane (PDMS), to replicate and transfer patterns. The process begins with the fabrication of a master mold, often created using photolithography or other precision machining techniques. This master mold defines the desired pattern, which is then transferred to a PDMS stamp or mold through casting and curing. The elastomeric stamp can then be used in various ways to transfer patterns to a substrate or create structures in materials [10,15].

Key techniques within soft lithography include microcontact printing (µCP), replica molding (REM), micromolding in capillaries (MIMIC), and microtransfer molding (µTM) [16]. For example, in microcontact printing, the PDMS stamp is “inked” with a functional material and pressed onto a substrate, leaving behind a patterned coating [17]. Alternatively, in replica molding, the PDMS mold is filled with a liquid precursor, which solidifies to form a patterned material [18].

As anticipated, one of the most significant applications of soft lithography is the fabrication of microfluidic devices. This process typically involves the following steps [10]:

1. Master Fabrication: A silicon or SU-8 mold is created using photolithography, where a photoresist layer is patterned with UV light to form microchannel structures on the substrate. The master serves as the template for subsequent PDMS molding;
2. Casting and Curing PDMS: Liquid PDMS is poured over the master mold and cured at an elevated temperature to solidify. Once cured, the PDMS is peeled off, forming a negative replica of the microchannel patterns;
3. Device Assembly: The PDMS replica is bonded to a flat substrate, often a glass slide or another PDMS layer. Bonding is typically achieved via plasma treatment, which activates the surfaces and enables strong adhesion. This creates enclosed microchannels within the device;
4. Integration and Functionalization: Additional components, such as inlet and outlet ports, are added to the device. The microfluidic channels can also be functionalized with coatings or molecules for specific applications, such as biological assays or chemical reactions.

Soft lithography’s advantages, such as its low cost, compatibility with diverse materials, and flexibility, make it ideal for prototyping and small-scale production [10,14,16]. However, its limitations in resolution, durability, scalability, and precision alignment make it less suitable for certain high-precision or large-scale applications [8]. Despite these challenges, its accessibility and adaptability have cemented its role as a powerful tool in modern microfabrication.

## 3. 3D Printing Technologies for Microfluidic Devices

Also known as additive manufacturing (AM), 3D printing is a technology which has been developed since the 1980s. It is a form of rapid prototyping which manufactures objects starting from 3D computer models, also known as computer-aided design (CAD) models, adding material one layer over the other. This type of process contrasts with traditional methodologies of subtractive production (e.g., milling machines or lathes), as the latter start from a block of material from which chips are mechanically removed [19]. The various types of AM differ mainly in how the layer is created with the different materials and how two consecutive layers bond together. These differences impact the accuracy of the final product as well as its overall properties [20,21,22].

Thanks to the possibility of creating any type of geometry which would otherwise be difficult to produce in a reasonable time and at a low cost, 3D printing finds application in many fields, including industry [23], architecture [24], agriculture [25], dentistry [26], aerospace [27], medical research [28], and various fields of engineering, including microfluidic engineering.

**Figure 2:**
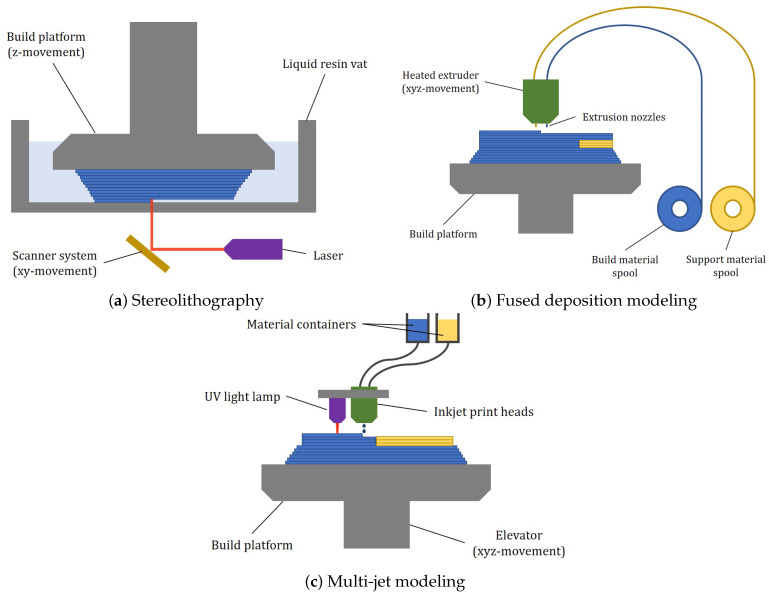
Schematic illustration of various 3D printing technologies.

Among the different types of 3D printing technologies, the ones used the most for the production of microfluidic devices are fused deposition modeling, multi-jet modeling, stereolithography, digital light processing, and direct laser writing. The last three fall under the category of technologies which rely upon photopolymerization for their functioning. Some schematic illustrations are reported in Figure 2. Selective laser sintering (SLS) is another 3D printing method which is widely used in biomedical engineering for the production of bone scaffolds, personalized implants, and biodegradable drug delivery devices [8,29]. It is, however, unsuitable for the fabrication of microfluidic devices since it involves sintering powdered materials, such as nylon [30], to create 3D objects, which are often hard to remove after the printing process, resulting in rough surface finishes and limited resolutions [8,29]. These surface imperfections can disrupt fluid flow in microfluidic channels and make it challenging to achieve the precise geometries and smooth channel walls required for accurate fluid control. For this reasons, SLS is not covered in this work.

### 3.1. Photopolymerization

Photopolymerization is a versatile 3D printing process which involves the use of light to initiate a chemical reaction, transforming liquid photopolymer resins into solid structures. This process relies on photoinitiators within the resin which absorb light at specific wavelengths, generating reactive species which trigger polymerization [31,32,33]. Several 3D printing technologies leverage photopolymerization, each distinguished by the method used to expose the resin and the achievable resolution. The most prominent technologies include stereolithography, digital light processing, and direct laser writing, which encompasses advanced methods such as two-photon polymerization.

Invented in 1986 by Chuck Hall [34], stereolithography (SLA) has become one of the most commercially viable and popular AM technologies available at present. Originally designed for use in manufacturing sectors, like the automotive and aeronautical industries, this technology has shown remarkable versatility, being capable of producing objects ranging from sub-micron to decimeter scales. Consequently, it has found applications in biomedical and translational research, enabling the fabrication of surgical tools, customized implants and prosthetics, biomaterial scaffolds and cellular scaffolds for tissue engineering, and microfluidic chips [22,35,36]. In order to create the desired object, stereolithography hardens polymer precursors in the form of resins through a focused UV laser. Upon UV light exposure, photoinitiator molecules generate free radicals and reactive species, triggering polymerization of the resin and forming a solid material. The first polymerized layer adheres to a build platform, which supports the structure during fabrication. After this layer is cured, the platform moves at a set distance along the z axis, and the process is repeated for the consecutive layers until the 3D structure is fully formed [37].

In the case of digital light processing (DLP), the focused laser used in SLA is replaced by a digital light projector, which cures an entire layer at once [38]. The digital light projector is based on micron-sized mirrors, each of them representing a single voxel. The number of micromirrors as well as the size of the build area determine the resolution of the final part, and since the projected light covers a complete layer at once, DLP printers generally have shorter printing times than SLA printers [38,39]. Since patent protection has expired and costs have become more affordable, DLP printers are currently emerging in the microfluidic field, being an attractive alternative to soft lithography due to the higher resolution the projector can utilize [40,41].

For a long time, SLA and DLP have been the main 3D printing technologies using resins. In recent years, however, new variations of these technologies have emerged, including liquid crystal display (LCD), which have further expanded the landscape of resin-based 3D printing. LCD 3D printing, also known as masked stereolithography apparatus (MSLA), is a resin-based additive manufacturing technology which utilizes an LCD screen to photopolymerize resins layer by layer [39,42]. As in SLA and DLP, a vat of liquid photosensitive resin is selectively exposed to UV light which, in this case, is emitted through a dynamic mask created by an LCD panel. The LCD screen controls the light pattern, curing only the areas required for each layer of the 3D model. As the printing progresses, the build platform incrementally moves, allowing the object to be constructed layer by layer with high precision and detail [42,43,44]. By curing an entire layer simultaneously rather than tracing it with a laser, LCD 3D printing achieves faster production speeds, particularly for multi-part prints or larger models, which are comparable to those reached with DLP printers. One of the main beneficial aspects of this technology is its cost-effectiveness, as it relies on consumer-grade LCD screens rather than specialized components [42,43].

Direct laser writing (DLW) is a fabrication technique which uses a focused laser beam to directly write patterns or 3D structures into a material. Pioneering work on single-photon DLW began in the early 1990s, and the first demonstration of multi-photon polymerization occurred in 1997. This breakthrough quickly gained traction in the photonics community due to its ability to fabricate intricate 3D nanostructures with high precision [45,46,47].

DLW encompasses a range of techniques, with some relying on processes like material ablation or deposition, but a significant subset focuses on photopolymerization [46,47], which is the primary technique discussed in this section. Photopolymerization-based DLW is particularly well suited for fabricating polymer-based micro- and nanodevices. DLW technologies which use one-photon absorption are typically employed for producing 2D patterns and require lower laser power. In contrast, two-photon absorption-based DLW is used to create 2D, 2.5D, and 3D structures of arbitrary shapes, offering higher resolutions as fine as sub-100 nm [46].

Considering the two-photon absorption process, two photons are absorbed simultaneously, in contrast to stereolithography, where only a single photon is absorbed. Since two-photon absorption is a nonlinear process, it confines the photopolymerization volume (i.e., the voxel) to the nanoscale range, resulting in a high resolution for the printed objects. To achieve two-photon polymerization, the intensity of the scanning laser is carefully controlled to exceed the threshold intensity for nonlinear absorption of the photopolymer material. The position and depth of the polymerization area are precisely controlled by a stage controller, allowing the focused laser beam to scan across the polymer layer. This process enables the creation of high-resolution microstructures of specific shapes, with unexposed areas being removed after development [46,48]. The high resolution, however, comes at the expense of the printing speed, which is quite slow, and the high costs related to the machine, resulting in the main factors slowing down the widespread of 2PP [8,48,49].

When analyzing the mechanical properties of objects printed though photopolymerization, strong covalent bonds can be found, giving an isotropic behavior to the prints, as well as high mechanical strength and the absence of leakage [50].

One of the adjustable parameters before printing is the thickness of the layers into which the CAD drawing is divided. For small layer thicknesses, the printed parts present a higher yield strength, tensile strength, and impact strength. This is because smaller layers create fewer and smaller voids. In contrast, larger layer thicknesses lead to the formation of bigger voids, which reduce the density of the printed part and result in a lower overall strength [19,51]. The thickness also influences the printing time, which becomes longer for smaller thicknesses. This causes longer irradiation of the layers, resulting in additional gain in terms of mechanical strength and Young’s modulus. Indeed, Cingesar et al. [52] found that mechanical properties are improved for longer curing times and increased post-curing durations, while considering the same post-curing treatment, the tensile strength increases, while the elongation at break decreases.

### 3.2. Fused Deposition Modeling

Developed in the late 1980s by Scott Crump and commercialized in 1990 by Stratasys [53], fused deposition modeling (FDM) produces objects using thermoplastics and finds applications in the modeling, prototyping, and manufacturing processes of various fields. The machinery features a heated nozzle into which polymer filaments are fed by an extruding motor. Once the material reaches its molten state, it is deposited onto a heated bed through extrusion forces applied by the nozzle, which can move in the *x*, *y*, and *z* directions, following a cross-sectional path [54,55]. Among the numerous materials used for FDM, nylon, polypropylene (PP), thermoplastic polyurethane (TPU), polyvinyl alcohol (PVA), and composite filaments can be found [56]. The print quality and mechanical properties of the final object are strongly dependent on the used process parameters [57,58]. Bakir et al. [58] provided an overview of these parameters, among which we can, for example, find the build direction and raster orientation and how they affect the morphology and strength of a printed part, finding that while the build direction has a noticeable influence on the strength of a part—vertically built specimens have lower flexural strengths than horizontally built specimens—the raster orientation does not have this effect but is rather an important parameter when considering crack propagation directions. Another important factor is the temperature of the nozzle [58,59,60]. Higher nozzle temperatures not only increase the crystallinity of the printed material but also enhance the mechanical properties, such as the tensile strength and Young’s modulus. This improvement can be attributed to the better rheological properties, including reduced viscosity, at elevated temperatures, which facilitate smoother material flow and better layer adhesion [58,59]. The viscosity of the polymer melt is temperature-dependent, meaning that at lower temperatures, the viscosity is relatively high, causing the extruding motor to apply excessive force on the material and potentially deforming the polymer chains. As the material exits the die, the chains attempt to recover their initial shape due to their viscoelastic nature, resulting in swelling of the extrudate [60].

### 3.3. Multi-Jet Modeling

Multi-jet modeling (MJM), also known as “PolyJet” or “photopolymer inkjet printing”, is a rapid prototyping technology which features multiple print nozzles and a UV light lamp. First developed in 1990 by the company Objet, later acquired by Statasys [8,61], this technology combines aspects of stereolithography and inkjet printing. UV-sensitive photopolymers together with wax material, used as support during building process as it fills voids and other non-free-standing features, are jetted onto a flat platform by multiple nozzles located on the print head. Photopolymers, in the form of monomers, are polymerized, thus transitioning from the liquid starting state to the solid final state, immediately after being deposited by means of UV lamps, which are usually located on the print head. At the end of each print, a post-build process is performed to easily remove the support material through manual peeling or soaking in sodium hydroxide (NaOH). This step does not affect the dimensional accuracy or surface finish, as happens in other 3D printing technologies [62,63]. The advantages of MJM are its high accuracy and good surface finish, the possibility to use multiple materials and colors for the same part, and easy removal of the support material [64]. Having chemical processes similar to stereolithography, the main properties of the created objects also largely correspond to those printed with SLA. However, when it comes to the use of multiple materials during a single print, the mechanical properties of the final object, in addition to depending on factors such as surface finishing, aging, and lighting conditions, also depend on the print orientation and proportions of the various materials [61,65]. Different combinations may lead to distinct localized fracture areas and failure, especially at the interface of two consecutive layers of different materials, where delamination usually happens [63]. The major obstacle preventing MJM from being widely used in microfluidic manufacturing can be identified with the support material which must be removed from the channels or otherwise obstructed [6,66]. This affects the final dimensions of the channels, which conventionally do not assume dimensions above 500 µm [6]. However, recent studies have managed to overcome this problem, printing channels smaller than 100 µm [66,67].

## 4. Comparison of 3D Printing and Soft Lithography

### 4.1. Cost

Although soft lithography is preferred due to the perceived high upfront costs of 3D printing (e.g., costs for a DLP printer typically range from USD 15,000 to 30,000 [43]), the latter tends to be more cost-effective in the long run, particularly when considering long-term production and scalability [29,68]. However, among the various 3D printing technologies, there are also those with more affordable machine costs. For instance, LCD printers have been gaining increasing popularity in recent years, largely due to their low prices, which range between USD 150 and 600 [43]. Shafique et al. [43] demonstrated that despite its low costs, this technology is well suited for high-resolution production of devices such as microfluidic chips with an embedded mixer, membrane microvalves, ELISA-on-a-chip capillaric circuits, and organ-on-a-chip devices, which have all been previously produced with other methods (laser machining, replica molding, and DLP 3D printing).

The manufacturing cost is not just given by the cost of machinery; it also includes the costs of labor, materials, and energy [69]. Being an automated process, 3D printing significantly reduces labor costs compared with the manual and labour-intensive nature of soft lithography. Indeed, the latter requires skilled technicians and longer production times, particularly for complex or multi-layered devices, driving up expenses [8,68]. Also, 3D printing is more efficient in terms of material usage and production. As an additive process, it minimizes waste and allows for simultaneous production of multiple copies, which reduces the cost per device. In contrast, soft lithography often involves more material as well as longer and more intricate fabrication steps, which increase overall costs [3]. Moreover, 3D printing offers scalability advantages, particularly with the growing availability of affordable desktop printers. These printers enable in-house production, bypassing the need for costly external services and allowing for low-cost, on-demand fabrication. Finally, it provides greater predictability and flexibility [68]. Its automated nature facilitates accurate cost estimation and allows for quick design changes without incurring significant cost increases, as happens with soft lithography, which sometimes leads to delays and costly redesigns.

Overall, while the initial cost per unit may be comparable, 3D printing tends to be more cost-effective than soft lithography over time, particularly as production volumes increase.

### 4.2. Geometrical Complexity

While soft lithography, particularly with PDMS, has been a keystone technology for microfluidics, it has significant limitations in terms of material versatility and complexity of achievable geometries. In contrast, 3D printing offers a flexible and powerful alternative, enabling the creation of complex, customized geometries and the use of a broad range of materials, overcoming the limitations of traditional methods.

Additive manufacturing enables the production of complex three-dimensional structures, which are often necessary for replicating the intricate microenvironments found in biological systems, enhancing control over cell and fluid dynamics [70]. On the contrary, soft lithography, which is generally confined to planar fabrication, struggles to achieve the necessary three-dimensionality required for advanced biotechnological applications.

Complex networks of microchannels have been successfully printed by the scientific community [71]. In the human body, blood channel sizes range from approximately 8 µm in capillaries to several millimeters in arteries, and cells typically range from a few microns to about 100 µm [72]. To recreate biological models, scientists often take inspiration from nature, biomimicking structures and patterns found in both the animal and plant worlds [73]. Vascular networks in nature often obey Murray´s law, according to which the optimal design which minimizes flow resistance while meeting diffusion requirements is given when the cube of the radius of a parent vessel is equal to the sum of the cubed radii of bifurcated child channels when flow is laminar and the volumetric flow rate is conserved [73,74,75].

**Figure 3:**
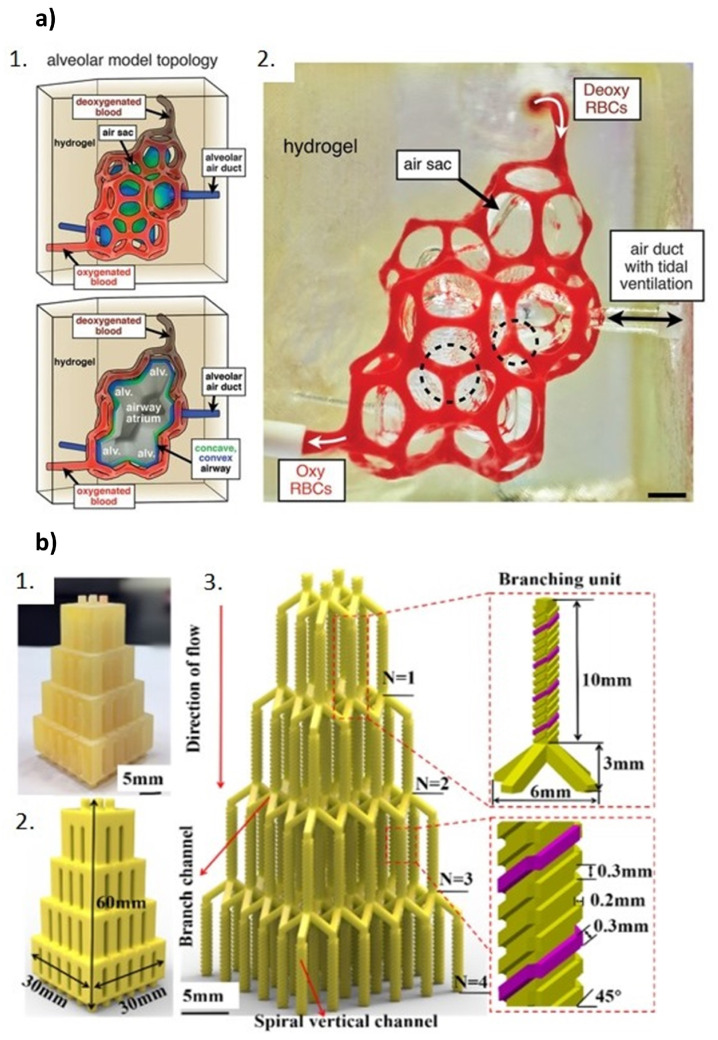
(**a**) Bioinspired alveolar model with ensheathing vasculature, based on 3D tessellations of the Weaire–Phelan foam topology: (1) architectural design and cutaway view and (2) photograph of the printed alveolar model in hydrogel. Reproduced from [76]. (**b**) Multi-drug combination microfluidic chip: (1) 3D printed prototype; (2) CAD model; and (3) illustration of the interconnected microchannel network with details of the branching structure. Reproduced from [77].

Grygorian et al. [76] managed through projection stereolithography to shape hydrogels into intricate and functional vascular architectures, comprising intravascular 3D fluid mixers and functional bicuspid valves. Printing of the hydrogels was made possible by the use of biocompatible food dye additives as photoinitiators. Furthermore, based on 3D tessellations of the Weaire–Phelan foam topology, the authors succeeded in the development of a bioinspired alveolar model with ensheathing vasculature, formed by 185 vessel segments and 113 fluidic branch points (Figure 3). This allowed them to study the oxygenation and flow of human red blood cells during tidal ventilation and distension of a proximate airway.

A three-dimensionally complex multi-drug combination microfluidic chip was printed by Chen et al. [77] (Figure 3). The microchannels possess compact helical structures which, by being connected to four inlets, promote rapid mixing of solutions, forming 36 combinations (given at the outlet) of different concentrations and ratios. Branching of the four inlet channels in the 36 outlets occurred at four levels. After printing via MJM, the device was tested for drug mixing of celecoxib, 5-fluorouracil, cyclophosphamide, and doxorubicin, and the combined effect of these drugs has been demonstrated by the viability test of A549 cells.

The creation of valves and pumps integrated into microfluidic devices is also facilitated by 3D printing, which are indispensable parts when dealing with automated handling of fluids. The manufacturability of these through soft lithography is a complex task, since the micrometrically sized parts need to be manually assembled, requiring adequate engineering expertise as well as expensive fabrication facilities [8,78,79]. Rogers et al. [79] demonstrated the successful fabrication of photopolymerized microfluidic devices with integrated valves, which was completed in under an hour. Horizontal flow channels with cross-sections of 350 µm × 250 µm and vertical channels 350 µm in diameter were printed with 100% yield. Valve diameters as small as 2 mm functioned as expected, with performance holding up through 800 actuations. However, the resin which was initially used resulted in devices which were not fully optically transparent and may have exhibited bulk fluorescence. Subsequently, by optimizing resin formulation [80], the group was able to reduce the dimensions of the valves by 90% with respect to their previous design, enhancing the durability to one million actuations [81]. The team also developed compact 3D-printed pumps, achieving flow rates up to 40 µL/min and characterized pump performance under different conditions. Additionally, a three-to-two multiplexer with an integrated pump was created, which can function as both a serial multiplexer and a mixer. Rapid prototyping allowed for design improvement, fabrication, and testing within a single day.

### 4.3. Materials

Material selection is another area where 3D printing excels. Soft lithography is primarily limited to PDMS, whereas 3D printing can utilize a diverse range of materials, including polymers, hydrogels, metals, and ceramics [70]. Before the advent of microfluidic chips, microchannels were used in gas chromatography in the form of glass capillaries [82]. Today, in addition to glass, which is preferred for optical measurements due to its high transparency, well-defined surface chemistry, and excellent resistance to high pressure [82], polymers such as polystyrene, polyvinyl chloride, polymethyl methacrylate, polyethylene glycol diacrylate, and polycarbonate are also commonly used, often in conjunction with 3D printing. These materials offer various properties such as low thermal conductivity, compatibility with biomedical applications, and the potential for surface modification [82]. Additionally, paper-based materials are also used in microfluidics for their inherent porosity and capillarity, which facilitate passive transport of liquids, as well as for the possibility to be functionalized through treatments with different liquids in order to modify the desired wettability, conductivity, mechanical resistance, and color [82]. In addition, 3D printing enhances the capabilities of paper microfluidics [83] by enabling the integration of solid support structures [84,85], scalable fabrication tools [86], and the direct printing of porous materials [87].

### 4.4. Transparency

**Figure 4:**
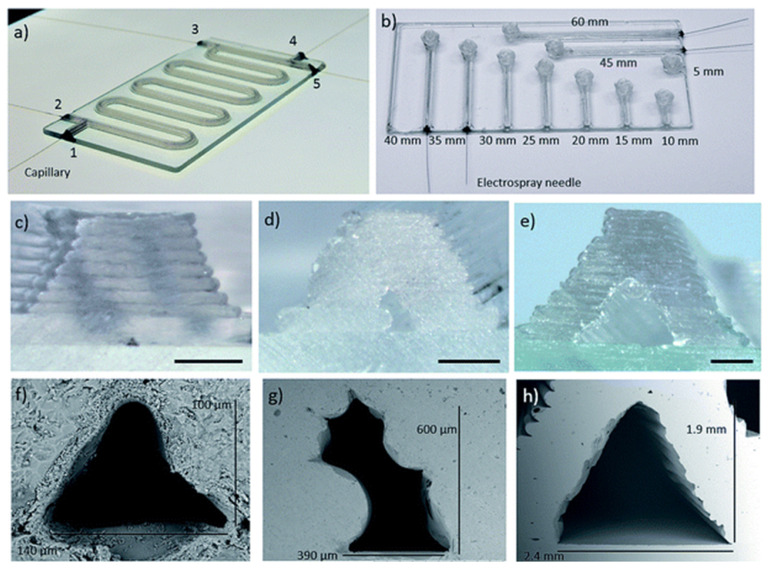
Glass microfluidic devices: (**a**) microreactor device with a 400 mm-long channel; (**b**) direct infusion device with channels lengths as indicated in the figure; (**c**–**e**) reported optical micrographs of the cross-section of three different-sized channels; and (**f**–**h**) their reported scanning electron micrographs. Reproduced from [89].

In terms of optical properties, transparency is crucial for applications requiring visual monitoring of fluid flow and cell interactions. Materials like glass are preferred for their excellent optical clarity, low fluorescence background, and surface stability, making them ideal for optical measurements [82,88]. Transparency also facilitates direct observation of how reagents interact within a device, which is essential for accurate diagnostics and research applications [3]. Recent advancements in additive manufacturing have expanded the possibilities of using glass for microfluidic devices, enhancing not only their optical properties but also the precision and speed of fabrication. For instance, Gal-Or et al. [89] demonstrated the potential of 3D printing with molten glass, enabling the production of complex microfluidic devices with channels as fine as 140 µm wide and 100 µm high (Figure 4). The technique they used allows for rapid manufacturing within minutes, followed by a short annealing process, significantly reducing production times compared with conventional methods. Moreover, this approach supports the creation of large, fully integrated devices with sizes up to 200 × 200 × 350 mm^3^, as well as channels which can extend up to 2.5 m in length, highlighting the ability to fabricate both intricate and large-scale structures efficiently.

Alternative methods have also been developed to produce transparent glass microstructures under milder temperatures. Li et al. [90] presented a photochemistry-based strategy using a photocurable PDMS resin which is 3D printed by 2PP and then converted into silica glass through deep ultraviolet (DUV) irradiation in an ozone environment. Ozone, which is given by the conversion of oxygen when hit with DUV irradiation, is responsible for conversion of the Si-C bonds found in PDMS into the Si-O-Si bonds found in silica. This process occurs at relatively low temperatures (~220 °C) and takes less than five hours, offering a milder alternative to traditional high-temperature glass manufacturing. The DUV-ozone conversion process ensures the formation of undistorted silica glass microstructures with linear shrinkage of approximately 24%, which is comparable to high-temperature sintering processes, maintaining the integrity of the printed parts without cracks or flaws and achieving a high-quality optical resolution comparable to state-of-the-art two-photon polymerization printings.

Similarly, Nguyen et al. [91] utilized a two-step process involving direct ink writing of colloidal silica suspensions in order to form porous, low-density green bodies, which are subsequently sintered into fully dense, amorphous glass structures at temperatures below the melting point of silica. This technique allows for the fabrication of sub-millimeter features without the need for high-temperature processing during the initial printing phase, resulting in higher-resolution structures due to thinner filament extrusion and controlled shrinkage during densification.

Furthermore, Kotz et al. [92] discussed the use of a nano-composite material given by amorphous silica nanopowders dispersed in a photocurable binder matrix, which was printed through stereolithography (SLA) or microlithography. After preliminary heat treatment at 600 °C to remove the binder, the remaining nanopowder was sintered at 1300 °C to form a highly transparent, full-density fused silica glass. This process allows for the rapid printing of microfluidic chips within 30 min, with heat treatment completed in about two days, offering a practical approach for producing transparent glass microstructures with even finer features (resolution of tens of microns with microlithography).

**Figure 5:**
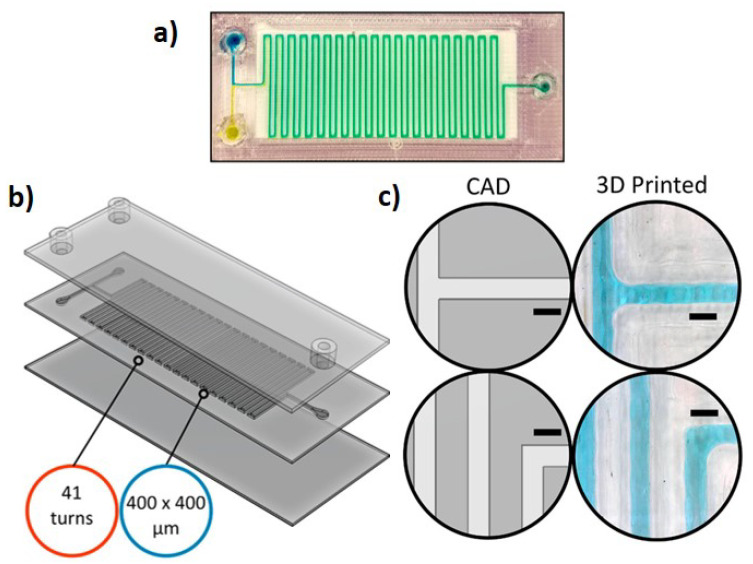
Transparent microfluidic device (**a**). Channels with a cross-section of 400×400 µm are 3D printed through FDM (**b**). However, due to the circular nature of the nozzle, the printing of 90° sharp angles is hindered (**c**). Reproduced from [97].

The Netherlands, Formlabs Clear V4 and BioMed Clear V1 (Formlabs, Somerville, MA, USA), VisijetCrystal (3D Systems, Rock Hill, SC, USA), Asiga PlasClear (Asiga, Alexandria, Australia), FunToDo NanoClear (FunToDo, Kotka, Finland), and MiiCraft BV007a Clear Resin (Miicraft, Hsinchu, Taiwan) can be found [93,94,95,96]. However, despite the popularity of photopolymerization, other 3D printing technologies, such as fused deposition modeling, have also been explored for producing transparent microfluidic chips. In FDM, the optical properties, such as the mechanical properties, are highly dependent on factors such as the nozzle temperature, print speed, and cooling rate, with transparency decreasing as the number of layers increases, while the likelihood of trapping air bubbles in the print also increases, causing light scattering [97]. Hence, although FDM may seem less suited for 3D printing of transparent objects due to its layer-by-layer extrusion process, researchers have made significant advancements in order to overcome this problem. Romanov et al. [97] managed to 3D print transparent channels with a cross-section of 400 µm × 400 µm (Figure 5) and perform droplet generation and tracking as well as DNA melting analysis, demonstrating that further annealing of the chips can also make them suitable for high-temperature applications.

**Figure 6:**
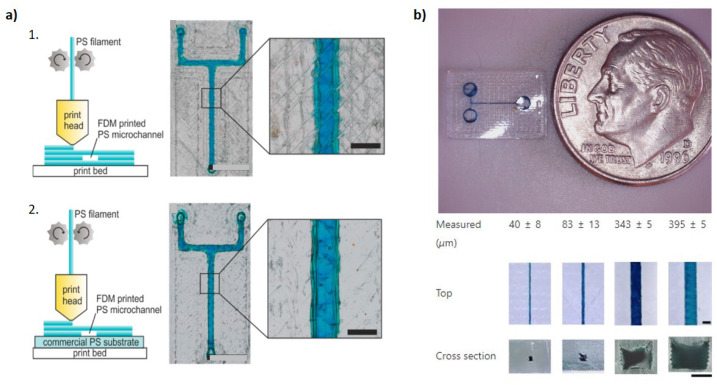
(**a**) Microfluidic devices with a Y channel are produced in PS through FDM. (1) PS filament is either deposited directly onto the print bed or (2) deposited on top of a commercial PS substrate. By performing printing on the substrate, the surface texture of the print bed is avoided, therefore enhancing the transparency of the microchannels (scale bar: 600 µm). Reproduced from [99]. (**b**) Single-channel microfluidic device 3D printed using TPU and pictured next to a US dime for reference. The smallest channel printed had a cross-section of ~40 µm. Reproduced from [100].

Quero et al. [98] systematically studied how parameters such as the extrusion flow rate and nozzle geometry influence transparency. The findings showed that transparency improves when the cross-section of extruded filaments is oblong, as this shape fills voids more effectively than circular filaments, enhancing layer bonding and reducing air gaps. Additionally, the oblong shape promotes better sintering between rasters and layers. As a result, microfluidic devices with 70 µm ± 11 µm-wide channels and transparency levels as high as 80% light transmittance were achieved, values which are comparable to glass and even surpass some SLA-printed devices. Transparent microfluidics were also successfully 3D printed through FDM with materials such as polystyrene (PS), reaching channels as small as 300 µm [99], and thermoplastic polyurethane (TPU), with channels as small as 40 µm [100]. Both are cost-efficient and biocompatible materials with excellent mechanical properties (Figure 6).

In printers which use photopolymerization technology, the wavelength of the emitted light plays a big role in printing transparent parts. These printers usually feature either a 405 nm or a 385 nm laser source, with the 405 nm option being more common due to the lower cost of the associated hardware [96,101]. To date, printers operating with 385 nm wavelengths are DLP-based. Among them, we can find, for example, the ones produces by Asiga, Genera3D and Origin [101]. Transparent resins are more efficiently patterned with a 385-nm light, and when cured at this wavelength, they tend to remain clear and non-yellow after printing [96,101]. Typically, 385-nm light enhances the z axis resolution in DLP printers, and in order to maintain this high resolution, manufacturers often reduce the overall print volume [8]. Being aware of which wavelength a photopolymerization printer performs with is especially important when printing objects designed with voids and channels, since the resin trapped in these spaces could still receive small quantities of light as the top plane layers are printed [8,102]. To overcome this issue, van der Linden et al. [102] performed wavelength selection on a DLP printer combined with a careful choice for the photo-absorber and photo-initiator in order to better control resin polymerization and improve the channel resolution. By doing so, they were able to obtain 260 mm-long channels with a 100×111 µm cross-section and 500 mm-long channels with a 150×148 µm cross-section and details below 100 µm. When considering the channel resolution, the viscosity of the material is also a critical factor. Indeed, low-viscosity resins are preferred since they are easier to manage and remove during the post-processing steps. Unremoved resin may be solidified during UV post-treatment, leading to inconsistent channel dimensions and compromising device functionality [3,72,103].

### 4.5. Biocompatibility

Biocompatibility remains a paramount concern, especially for microfluidic devices intended for cell studies. By definition, biocompatible materials must be engineered to interact with living tissues without causing adverse reactions, and they must meet stringent standards such as ISO 10993 in Europe and USP Class VI in the United States [104,105]. However, the post-processing conditions, including curing, exposure to UV light, and chemical treatments, can significantly affect the biocompatibility of parts which may have been printed with resins marketed as biocompatible. Excessive UV exposure, for instance, can lead to photo-oxidation, causing degradation in the material’s properties, such as its gloss, flexibility, and overall performance [106]. Moreover, biocompatibility can vary widely among objects printed with the same material but with different technologies, resulting in some materials demonstrating significant toxicity unless properly treated [105]. This phenomena was studied by Alifui et al. [105], who evaluated through the OECD fish embryo test the toxicity of materials printed with stereolithography and material jetting. They tested four materials—two MED (Objet/Stratasys, Eden Prairie, MN, USA) resins and two Visijet (3D Systems, Rock Hill, SC, USA) resins—in both untreated and ethanol-treated forms. Generally, untreated materials were found to be unsafe, with the Visijet materials performing better than the MED materials. Ethanol treatment improved the biocompatibility of the MED materials but produced inconsistent results with the Visijet materials. Notably, untreated Visijet Clear was safer than its treated form, while treated Visijet Crystal showed severe toxic effects, leading to test terminations due to high toxicity despite initially high survival rates for the fish embryos. Moreover, the authors addressed various strategies to improve the biocompatibility of photopolymers, including heat treatment in a nitrogen atmosphere, supercritical CO_2_ extraction, and post-curing with isopropanol, as some treatments like ethanol were found to be ineffective in other studies as well. The inconsistency in results underscores the necessity for ongoing biological assessments, particularly for photopolymers intended for clinical use, as reliance on in vitro tests alone may not accurately predict clinical outcomes. Guttridge et al. [104] also highlighted the lack of standardization in the amount and quality of information provided with commercially available 3D printing materials. They emphasized that users should exercise due diligence when selecting materials for their specific applications.

In response to this lack of standardization and transparency, several research groups have developed custom biocompatible resins specifically tailored for biological applications. These resins offer greater control over both biological and mechanical properties, overcoming the limitations of commercially available materials. For example, Warr et al. [107] introduced a non-cytotoxic 3D printing resin based on poly(ethylene glycol) diacrylate (PEG-DA) with avobenzone as a UV absorber, which is safer for cell-based applications than the commonly used 2-nitrophenyl phenyl sulphide (NPS). This avobenzone-PEG-DA resin supports high-resolution microstructures and can be used in applications like spheroid formation and cell migration, while cell adhesion can be enhanced after plasma treatment.

Another group formulated a biocompatible resin from low-molecular-weight PEG-DA (MW 250) and Irgacure-819 for stereolithographic printing of biomicrofluidic devices [96]. The resin enables a high Z-resolution due to strong UV absorption, and after UV post-curing in a water bath to remove toxic leachates, it is able to support long-term cell culture, including sensitive neurons. However, in comparison with PDMS, PEG-DA is not gas-permeable, therefore necessitating alternative strategies, such as perfusion, for effective cell culture in enclosed microchannels.

Further advancements include the development of a high-resolution 3D printing approach for microfluidic components, namely using avobenzone-based PEG-DA resin optimized through spectral engineering of the 3D printer’s optical source [108]. This method enables the fabrication of durable components such as pillar arrays, valves, and pumps, with applications extending to cell-based microfluidics, including cell chemotaxis experiments.

In addition to PEG-DA-based resins, polymethylmethacrylate (PMMA) has also been explored due to its optical transparency, low autofluorescence, and high biocompatibility. While traditionally shaped through injection molding, hot embossing, or subtractive processes, one study demonstrated that PMMA microfluidic chips can be directly 3D printed using FDM, achieving a minimum channel width of 300 µm, as well as comparable biocompatibility and hydrophilic properties to commercial PMMA [109].

In conclusion, 3D printing offers a versatile and powerful platform for the fabrication of microfluidic devices, providing significant advantages over traditional soft lithography. The ability to create intricate geometries, utilize a wide range of materials, and tailor device properties to meet specific application needs positions 3D printing as a superior choice for advancing microfluidic technologies. As discussed, 3D printing enables the fabrication of complex structures which may not be achievable with soft lithography while also accommodating a variety of materials, including those with enhanced mechanical, optical, or biocompatible properties. From a cost perspective, 3D printing is often seen as a more cost-effective solution, especially for high-volume production or rapid prototyping. However, it is important to acknowledge that certain 3D printing techniques, particularly those using photopolymerization technologies, may incur significant costs due to pre- and post-treatment processes. These steps, which often involve the removal of chemical residues and polymer leachates from UV curing, can be time-consuming and are dependent on the know-how of each research group. In some cases, these additional steps may negate the perceived cost benefits of 3D printing, particularly when biocompatibility is a concern. Thus, while 3D printing holds great promise, careful attention must be paid to material selection, the complexity of post-processing methods, and the potential impact on biocompatibility, which are all crucial factors in determining its long-term success in microfluidic device fabrication.

## 5. Future Developments and Trends

As the field of 3D printing for microfluidic devices continues to evolve, several key areas are positioned for significant growth and innovation. These developments have the potential to address the current limitations of the technology while opening new avenues for advanced applications in biology, chemistry, medicine, and engineering.

### 5.1. Technical Improvements in Printing Resolution and Speed

One of the foremost areas for future development in 3D printing of microfluidics is improving the resolution and speed of the fabrication process while being economically affordable. While techniques such as two-photon polymerization offer high precision at the microscale, they are limited by their elevated costs as well as slow printing speeds, making them unsuitable for large-scale designs [8,48,49]. Future research may focus on enhancing the scalability of high-resolution techniques through multi-beam approaches or advanced scanning strategies. Parallelized printing strategies could also significantly increase throughput without sacrificing precision [6,110].

Additionally, the refinement of hardware and software will play a pivotal role in advancing microfluidic chip production. Algorithms optimized for printing complex microchannel networks, alongside improved hardware which minimizes distortions, could enable the fabrication of highly intricate designs at faster speeds, further expanding the usability of 3D printing in microfluidics.

### 5.2. Development of New Materials for Specialized Applications

The development of new 3D printing materials which are better suited for specific microfluidic applications is another critical area for future exploration. While current polymers and resins used in 3D printing offer adequate properties for general use, they often fall short in terms of chemical resistance, optical transparency, and biocompatibility, especially for biomedical applications.

To address this, new materials which exhibit superior mechanical properties, such as increased elasticity, strength, and durability, need to be developed. These materials must also be customizable for different applications, including drug delivery, cell culturing, and organ-on-a-chip systems, which require materials that support cell growth and biological interactions. Biodegradable and bioresorbable materials could play a crucial role in medical devices and tissue engineering applications, where the need for temporary structures is critical [111,112].

Moreover, the ability to integrate multi-material printing will enable the creation of hybrid devices which combine rigid and flexible components, electrical functionality, or even sensors directly into the microfluidic architecture. Such advancements will enable more complex, functional, and application-specific devices, enhancing the capabilities of 3D printing even more.

### 5.3. Process Standardization and Regulatory Frameworks

Despite the potential of 3D printing technologies in microfluidics, the field is still hindered by the lack of standardized processes and regulations, particularly regarding material biocompatibility and sometimes reproducibility [113]. As the technology advances, ensuring that 3D-printed microfluidic chips meet industry standards for quality, precision, and safety will become essential, especially in the context of medical diagnostics, drug testing, and personalized medicine.

The establishment of clear guidelines for the certification of 3D printing materials, particularly in terms of biocompatibility, is crucial for the integration of these devices into clinical and commercial settings. Regulatory bodies such as the FDA and ISO may need to adopt specific standards for 3D-printed microfluidics to ensure that the materials used do not cause adverse biological reactions, especially for in vivo applications. This includes rigorous testing for cytotoxicity, immunogenicity, and long-term biocompatibility.

In addition to material safety, process standardization must focus on the reproducibility and reliability of 3D-printed microfluidic devices. Current fabrication processes can result in slight variations in the dimensions or surface characteristics of the microchannels, which could affect fluid dynamics and device performance [114]. Standardizing printing protocols, calibration procedures, and post-processing steps (e.g., cleaning and surface treatments) would mitigate these inconsistencies, ensuring that devices produced across different labs or industries yield comparable results.

### 5.4. Bioprinting and Microfluidics: The Future of Organ-on-a-Chip Devices

A promising trend at the intersection of 3D printing and microfluidics is the development of bioprinting techniques for organ-on-a-chip devices [115]. Organ-on-a-chip platforms are primarily used for mechanistic studies and proof-of-concept drug testing, often requiring sophisticated microfabrication processes. Bioprinting technology offers the potential to automate these processes, addressing challenges related to throughput and reproducibility in traditional organ-on-a-chip systems. These models typically incorporate 3D microchannels and complex structures to mimic the architecture of real tissues and organs, but controlling the properties and microstructure of soft scaffolds remains a challenge. Bioprinting can overcome this by allowing precise tuning of the mechanical properties, porosity, and microstructure of hydrogel scaffolds. The technology uses bioinks—soft biomaterials mixed with living cells—such as gelatin, alginate, and collagen, which are critical for fabricating functional tissue constructs [116]. Different bioprinting techniques, including extrusion, inkjet, stereolithography, and laser-assisted bioprinting, enable the creation of complex, biomimetic microenvironments which replicate human physiological conditions [116]. By combining bioprinting with organ-on-a-chip technology, researchers can create highly accurate in vitro models for drug testing, disease studies, and pharmacological modulation, enhancing the relevance and functionality of these systems for biomedical research [115,116,117]. Furthermore, the integration of vascular networks and other complex structures within microfluidic chips will enhance the functionality of organ-on-a-chip devices, accelerating the shift toward fully integrated systems which replicate human organ function, ultimately reducing the reliance on animal testing, and improving the predictability of clinical outcomes [115,117,118].

### 5.5. Sustainability and Environmental Considerations

With the rapid growth of 3D printing, there is also an increasing awareness of the impact this technology has on the environment. Many studies have been published over the years analyzing the environmental impact of additive manufacturing through different methods, such as life cycle assessment and design for environment [69,119,120,121,122]. However, due to the lack of adequate and standard metrics in analyzing this aspect, many works report conflicting results, sometimes indicating AM as a sustainable production method and sometimes indicating that it has no environmental benefit [122]. The field of green microfluidics is in its early stages, with a limited number of researchers seeking methods to produce microfluidic chips in an eco-friendly manner [88]. Future research should therefore focus more on finding and creating sustainable materials, such as recyclable and renewable polymers, thus reducing the impact of 3D printing processes, as well as introducing standardized evaluation methods.

## 6. Conclusions

In the last decade, 3D printing has emerged in numerous fields, including microfluidic engineering, becoming a powerful alternative to traditional soft lithography and offering significant advantages in terms of design flexibility, customization, cost-efficiency, and rapid prototyping. Despite still having challenges such as material limitations, biocompatibility concerns, and the need for higher resolutions and faster printing speeds, many researchers in the fields of materials science and printing technology are working to find solutions to these limitations. As the field progresses, in order to see a wider spread of 3D-printed microfluidics applications, improvements in process standardization and the establishment of clear regulatory guidelines will be crucial, particularly in areas like biomedical research, diagnostics, and drug development. By combining all of these innovations, 3D printing will most likely prevail among the technologies used for the creation of microfluidic devices.
